# Drug Targeting of Genomic Instability in Multiple Myeloma

**DOI:** 10.3389/fgene.2020.00228

**Published:** 2020-04-09

**Authors:** Meral Beksac, Sevinc Balli, Dilara Akcora Yildiz

**Affiliations:** ^1^Department of Hematology, School of Medicine, Ankara University, Ankara, Turkey; ^2^Kars Selim Public Hospital, Internal Medicine, Kars, Turkey; ^3^Department of Biology, Science & Art Faculty, Burdur Mehmet Akif Ersoy University, Burdur, Turkey

**Keywords:** genomic instability, DNA repair, multiple myeloma, molecular targets, small molecule inhibitors, PARP inhibitors

## Abstract

Genomic instability can be observed at both chromosomal and chromatin levels. Instability at the macro level includes centrosome abnormalities (CA) resulting in numerical as well as structural chromosomal changes, whereas instability at the micro level is characterized by defects in DNA repair pathways resulting in microsatellite instability (MIN) or mutations. Genomic instability occurs during carcinogenesis without impairing survival and growth, though the precise mechanisms remain unclear. Solid tumors arising from most cells of epithelial origin are characterized by genomic instability which renders them resistant to chemotherapy and radiotherapy. This instability is also observed in 25% of myeloma patients and has been shown to be highly prognostic, independently of the international staging system (ISS). However, a biomarker of aberrant DNA repair and loss of heterozygosity (LOH), was only observed at a frequency of 5% in newly diagnosed patients. Several new molecules targeting the pathways involved in genomic instability are under development and some have already entered clinical trials. Poly(ADP-ribose) polymerase-1 (PARP) inhibitors have been FDA-approved for the treatment of breast cancer type 1 susceptibility protein (BRCA1)-mutated metastatic breast cancer, as well as ovarian and lung cancer. Topoisomerase inhibitors and epigenetic histone modification-targeting inhibitors, such as HDAC (Histone Deacetylase) inhibitors which are novel agents that can target genomic instability. Several of the small molecule inhibitors targeting chromosomal level instability such as PARP, Akt, Aurora kinase, cyclin dependent kinase or spindle kinase inhibitors have been tested in mouse models and early phase I/II trials. ATM, ATR kinase inhibitors and DNA helicase inhibitors are also promising novel agents. However, most of these drugs are not effective as single agents but appear to act synergistically with DNA damaging agents such as radiotherapy, platinum derivatives, immunomodulators, and proteasome inhibitors. In this review, new drugs targeting genomic instability and their mechanisms of action will be discussed.

## Introduction

The 2015 Nobel Prize in chemistry was awarded to three scientists – Paul Modrich, Aziz Sancar and Thomas Lindahl, for their seminal studies into the mechanisms of DNA repair. They made pivotal contributions in the fields of carcinogenesis and of drug resistance mechanisms in malignancies including myeloma. The underlying causes of the emergence of multiple subclones in MM remain unclear. Genomic instability is one of the hallmarks of myeloma, and is present in both at the early stages of myeloma, and as the disease evolves under selection pressure. This clonal evolution is cited as an example of Darwinian behavior due to intrinsic properties of the tumor, its treatment and micro-environmental influences. Alternatively, it has been proposed that multiple subclones exist from the outset and that external factors favor the dominance or disappearance of individual subclones. Either way, it is evident that myeloma genomes are subject to dynamic evolution.

Hyperdiploidy is recognized to be one of the initiating genetic abnormalities in MM. Almost all the genetic abnormalities observed at diagnosis with the exception of RAS and MYC mutations are also detected at a similar frequency in patients with monoclonal gammopathy of undetermined significance (MGUS), and smoldering multiple myeloma (SMM). RAS and MYC mutations are both secondary events, MYC mutations seen in 55% of cases being the most frequent abnormality in MM (Walker et al., 2015). MYC deregulation is more or less ubiquitous and is mediated by non-physiological DNA damage and repair pathways (Affer et al., 2014). Similarly, deletions are also secondary genetic aberrations. Tumor suppressor gene loss or mutations such as Retinoblastoma may be driver events as Rb is located on the frequently deleted chromosome 13. 17p deletion/mutation is associated with a poor prognosis and is observed at increasing frequencies with each relapse. The relative frequencies of genetic abnormalities at diagnosis and at relapse cannot be interpreted to be direct evidence of clonal evolution. The pivotal study by Keats et al. was the first to report the different dynamic patterns of clonal genetic composition in 28 myeloma patients who were followed for up to 65 months. They identified three scenarios: one-third of patients displayed stable genomes over time and this was highly associated with low-risk hyperdiploid disease; another one-third of patients were found to have clonal heterogeneity at diagnosis but with the later reappearance of regions previously considered to be biallelic deletions; finally, the remaining one-third of patients had a pattern consistent with linear evolution. There was therefore evidence to support both genomic instability and clonal evolution.

The intra-clonal heterogeneity illustrated by the presence of subclones with distinct genetic mutations within the tumor population provides a rationale for the use of drugs in combination, rather than sequentially, in order to target eradication of minor as well as the dominant subclones. In addition, the concept of clonal tides according to the type of competing clones vary in dominance under selective therapeutic and environmental pressures, which can support the re-use of drugs that have been previously ineffective. Loss of heterozygosity (LOH) is a hallmark of genomic instability. The Dana-Farber Cancer Institute (DFCI) group was among the first to perform a longitudinal study of LOH in patients with myeloma. In one patient, they observed the acquisition of new genomic changes on chromosomes 3 and X over the course of a year. They were also able to induce resistance to corticosteroids *in vitro* following induction of homologous recombination (HR) using nickel, thereby demonstrating that DNA repair defects are involved in the acquisition of drug resistance.

Although high-dose melphalan continues to be an important drug in the treatment of MM, its role in inducing genomic instability as an off-target effect remains under debate. It is clear that secondary primary malignancies are more frequent in autologous stem cell transplantation (ASCT) recipients than in those who were not transplanted (Walker et al., 2015). In this regard, a recent study of genomic copy number alterations (CNAs) in a myeloma patient with the t(4;14) translocation, who was sequentially exposed to several drug classes (IMiDs, proteasome inhibitors and alkylating agents) found that genetic alterations occurred most frequently following exposure to alkylating agents (Walker et al., 2015). This observation was interpreted as raising the possibility of an increased susceptibility to genomic instability in cytogenetically defined high-risk MM and the potential harmful effects of DNA damaging agents in this subgroup of MM patients. This topic was extensively assessed in a previous review of genomic instability in myeloma (Gourzones-Dmitriev et al., 2013).

## Prognostic Role of DNA Repair Defects and Genomic Instability

Kassambara et al. developed a panel of DNA repair genes to assess their therapeutic role in patients included in clinical studies in the United States and in Germany. This panel included a total of 22 prognostic genes with five genes coding for Non-Homologous End Joining (NHEJ) (three bad: WHSC1, RIF1, XRCC5(KU80) and two good: PNKP,POLL), six genes for HR (five bad: EXO1, BLM, RPA3, RAD51, MRE11A and one good: ATM), three genes for FA (all of them bad: RMI1, FANCI and FANCA), eight genes for Nucleotide Excision Repair (NER) (six bad: PCNA, RPA3, LIG3,POLD3, ERCC4, POLD1 and two good: ERCC1 and ERCC5), two genes for Mismatch Repair (MMR) (both of them bad: EXO1 and MSH2) and one bad gene for Base Pair Excision Repair (BER) (LIG3) pathways. The DNA repair score was developed by a German group and was validated in the Total Therapy-2 studies. It was found to have a prognostic value independent of international staging system (ISS) and fluorescence *in situ* hybridization (FISH). The authors claim this DNA Repair (DR) score has the potential to identify patients whose tumor cells are dependent on specific DNA repair pathways. Recognition of such patients, might inform the design of treatments able to induce synthetic lethality through addiction to dysregulated DNA repair (Kassambara et al., 2015). Drugs with such potential include DNA-PKs inhibitors (NHEJ), RAD51 (HR), PARP1/2 (HR, alt NHEJ, BER), CHK2 (HR, alt NHEJ), and CHK1 (HR, NER) (Shaheen et al., 2011). These targeted drugs are today under clinical investigation in many cancers including MM.

Centrosomes, microtubule-organizing centers, play an essential role in the maintenance of dual spindle poles which are central to the accurate separation of genetic material into daughter cells during cell division. Centrosome amplification (CA) resulting in more than two centrosomes contributes to genomic instability and is common in cancer cells. CA is recognized to occur in MM cells and may have a role in disease progression (Chng et al., 2006). Based on gene expression data, a high centrosome index, closely associated with CA, was found to be a powerful independent prognostic factor in MM (Chng et al., 2008). Importantly, the centrosome index genes are involved in both centrosome duplication and function as well as in DNA repair; these include ATM, ATR, RAD51, XRCC2, and BRCA2. Dementyeva et al. (2010) found CA to be more frequent in B cells from MM patients when compared to those from healthy individuals. They also reported on the prognostic significance of the number of CA abnormalities and on the expression of centrosomal genes which were found to be downregulated in newly diagnosed (ND) patients, compared to relapsed patients (Dementyeva et al., 2013). In their study, ND MM patients with CA had a better prognosis compared to the CA negative group, indicating the clinical significance of centrosome clustering. Because CA leads to spindle multipolarity and subsequent apoptosis, cancer cells cluster their centrosomes into two functional mitotic spindle poles to avoid apoptosis (Quintyne et al., 2005). The pharmacological inhibition of genes involved in centrosome clustering which are included in the centrosome index including PARP, Aurora kinases or kinesin spindle proteins might therefore represent a promising approach in the treatment of MM. This is discussed further below.

## Targeting DNA Repair Defects

Malignant cells may show high level of genomic instability, stalled replication forks and Double Strand Breaks (DSB) leading to impaired DNA repair. This presence of these abnormalities in cancer cells led to the development of PARP-1 inhibitors as a new class of anti-cancer therapy. Homozygous loss of BRCA1 or BRCA2 predisposes women to breast and ovarian cancer and their function in HR-mediated repair is thought to be one of the major mechanisms by which they suppress tumor development. By restoring DNA repair and drug sensitivity, PARP inhibitors have shown clinical efficacy and have been approved for the treatment of solid tumors (Neri et al., 2011). Additional alterations in DNA repair pathways contribute to acquired drug resistance in many cancers including MM. The Fanconi anemia/BRCA (FA/BRCA) DNA damage repair pathway plays an important role in the cellular response to replicative stress induced by DNA alkylating agents such as melphalan. Significant downregulation of several DNA glycosylases (UNG2, NEIL1, and MPG) was also observed in MM cells resistant to the alkylating agent, melphalan, and was associated with increased efficiency of single strand or double strand break repairs (DSBs) (Neri and Bahlis, 2013).

## Clinical Implications of Novel Drugs Targeting DNA Repair and Genomic Instability in MM

### PARP Inhibitors as a Single Agent or in Combination With Proteasome Inhibitors, Alkylators

It is possible to target the mechanisms leading to genomic instability in MM. While genomic instability favors transformed cells by conferring a growth advantage and by allowing for the development of drug resistance, it also leads to targetable vulnerabilities. PARP1-2 inhibition results in DSBs and stalled replication forks in dividing cells and error-prone repair of these breaks, leads to cell death. High-throughput studies have shown that, in addition to the bi-allelic loss of BRCA genes, loss of function in other HR-related genes (including RAD51, ATR, PCNA, etc.) confers increased sensitivity to PARP inhibitors as they are unable to deal with the increase in lethal DSBs associated with replication fork collapse (Lord et al., 2008; Neri et al., 2011).

Nowadays, PARP inhibitors are approved as single agents in the treatment of ovarian cancer and BRCA-associated breast cancers. Alkylating agents, topoisomerase I inhibitors and platinum-based drugs have also been combined with PARP inhibitors to overcome DNA repair and increase efficacy (Patel et al., 2019).

**FIGURE 1:**
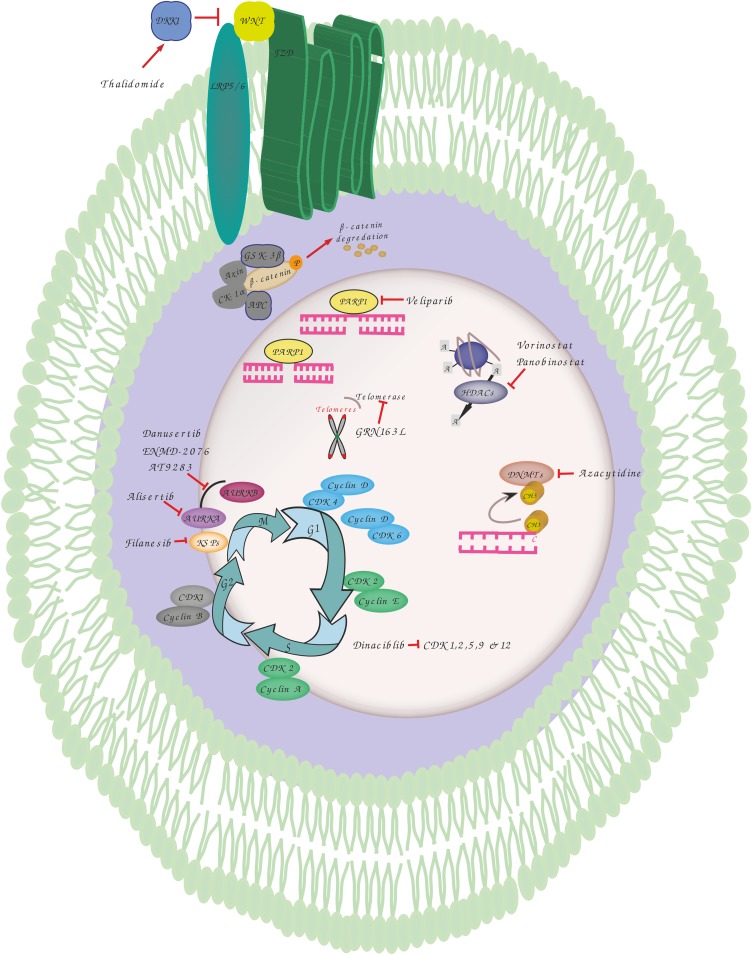
Overview of genomic instability targets and relevant drugs. Thalidomide induces dickkopf WNT signaling pathway inhibitor 1 (DKK1) that blocks the interaction between frizzled (FZD) receptors and lowdensity lipoprotein receptor-related protein 5 (LRP5) resulting in phosphorylation, ubiquitination and proteasomal degradation of b-catenin by destruction complex including adenomatosis polyposis coli (APC), glycogen synthase kinase 3 (GSK3), Axin and casein kinase 1 alpha (CK1α). Veliparib inhibits poly(ADP-ribose) polymerase 1 (PARP1) involved in various DNA repair pathways and in the maintenance of genomic stability. Vorinostat and Panobinostat are inhibitors of histone deacetylases (HDACs) that catalyze the removal of the acetyl moiety from the lysine residues of histones and non-histone proteins. Azacytidine is used to inhibit the activity of DNA methyltransferases which catalyze DNA methylation of cytosine resulting in transcriptional inhibition and gene silencing. GRN163L is an inhibitor of telomerase which prevents the shortening of telomeres length. Dinaciblib inhibits the activity of cyclin-dependent kinase (CDK) 1, 2, 5, 9, and 12 that play essential roles in cell cycle regulation. Filanesib inhibits kinesin spindle protein (KSP) which is important for the proper separation of spindle poles during mitosis. Alisertib (MLN8237) is a selective aurora A kinase (AURKA) inhibitor, while Danusertib, ENMD-2076 and AT9283 act by inhibiting both AURKA and B that have essential roles in mitosis.

When compared to normal tissues or other tumors, MM cells are known to be highly sensitive to proteasome inhibition. While this sensitivity to proteasome inhibitors is thought to be related to the high baseline level of protein (immunoglobulin) synthesis in plasma cells resulting in a “high proteasome load” and induction of the ER stress response, Neri et al. have suggested that proteasome inhibitors may also impair the ability of MM cells to repair damaged DNA. This phenomenon may well be myeloma’s Achilles’ heel. They were able to demonstrate in a SCID MM model that proteasome inhibition induces a functional “BRCAness” state by impairing the supply of BRCA1 and RAD51 to sites of DNA damage. The combination of proteasome and PARPi may therefore lead to synthetic lethality within plasma cells. The safety and efficacy of such approach was tested in a Phase I clinical trial (Neri et al., 2011). After administering oral Veliparib in combination with bortezomib and dexamethasone to heavily pre-treated patients with relapsed myeloma, they found this combination to be well tolerated and to have significant anti-tumor activity (Table 1). *In vivo* inhibition of PARP1-2 activity in MM cells has therefore been demonstrated (Figure 1). Further studies are ongoing to determine the maximal tolerable dose (MTD) of the different drugs in this regimen.

**TABLE 1:** Drugs Targeting of Genomic Instability in Clinical Trials in MM.

**Targets**	**Mechanism**	**Drugs**	**Studies**
PARP (poly(ADP-ribose) polymerase)	Induces to DSBs and stalled replication forks in dividing cells	Veliparib	Neri et al., 2011; Patel et al., 2019
CDK (cyclin-dependent kinases)	PARP1/2 sensitizies	Dinaciblib	Alagpulinsa et al., 2016
MALAT (Metastasis Associated Lung Adenocarcinoma Transcript)	MALAT1 RNA by RNase H using anti-sense gapmer DNA oligos in MM cells stimulated poly-ADP-ribosylation of nuclear proteins	Anti-MALAT1	Hu et al., 2018
1q12 region DNMT (DNA methyltransferases)	DNA methylation inhibitor provides evidence that site-specific hypomethylation of the 1q12 region	5-Azacytidine	Sawyer et al., 2015
HDAC (histone deacetylase)	Induce growth arrest and apoptosis	Vorinostat Panobinostat	Amodio et al., 2012
AURKA (aurora kinase A)	Induce G2/M cell cycle arrest	Danusertib	Lind et al., 2019
		ENMD-2076 AT9283	Shi et al., 2007; Hay et al., 2016
		Alisertib	Görgün et al., 2010
		Barasertib	Evans R. P. et al., 2008
KSP (kinesin spindle proteins)	Leads to metaphase arrest	Filanesib	Shah et al., 2017
WNT/B catenin	Inhibiting the proliferation of MM cells	CGK012	Choi et al., 2017
		BC2059	Savvidou et al., 2017
		Griseofulvin	Kim et al., 2011
NER (Nucleotide Excision Repair)	ERCC3 knock-down/NER deficiency led to a significant increase in sensitivity to melphalan	Spironolactone	Szalat et al., 2018; Alekseev et al., 2014
Telomerase	Blocks the template zone of telomerase	GRN163L	Schrank et al., 2018; Shammas et al., 2008

Alkylating agents such as busulfan used in transplant conditioning regimens, impair replication forks by DNA strand cross-linking, Neri et al. hypothesized that PARP inhibition with veliparib in combination with busulfan might lead to synergistic cytotoxicity against tumor cells in a xenotransplant model of myeloproliferative disease. In this study, vehicle- and veliparib-treated mice showed a similar median survival of 39 and 40 days, respectively. The combination regimen, however, increased median survival from 47 days (busulfan only) to 50 days (*P* = 0.02). Finally, they tested the combined effect of busulfan and veliparib on CD34+ cells obtained from the bone marrow or peripheral blood of five patients with JAK2V617F-mutated and two patients with CALR-mutated Myelofibrosis (MF). MF cell colony formation was further decreased when treated with the combination compared to busulfan alone (87% versus 68%; *P* = 0.001). In contrast, treatment of normal CD34 + cells with veliparib did not affect colony growth. They were therefore able to confirm the *in vitro* synergistic cytotoxicity of the PARP-1 inhibitor, veliparib, and busulfan (Patel et al., 2019). To the best of our knowledge, an alkylator-PARP inhibitor combination has yet to be tested in MM.

### Cyclin Dependent Kinase Inhibitors

MM cells are characterized not only by chromosomal instability but also by the dysregulation of upstream modulators of HR such as Cyclin-dependent kinases (CDK). The dysregulation and inhibition of CDKs in MM was recently reviewed by Maes et al. (2017). A CDK inhibitor, Dinaciclib, is known to reduce expression and to block phosphorylation of certain HR repair genes including Rad51 and BRCA1 (Figure 1). This impairment of HR repair sensitizes MM cells to the PARP1/2 inhibitor, ABT-888. Combined treatment with dinaciclib and ABT-888 *in vitro* has been shown to induce synthetic lethality in MM cells while normal CD19(+) B cells were spared (Table 1). These findings support the further assessment of dinaciclib in combination with PARP inhibitors in clinical trials in MM (Alagpulinsa et al., 2016).

### Antisense Oligomers

Metastasis Associated Lung Adenocarcinoma Transcript 1 (MALAT1) is a long non-coding RNA (lncRNA) which is expressed in normal tissues. MALAT1 is involved in the alternative non-homozygous end joining (A-NHEJ) pathway by binding to PARP1 and LIG3, two key components of the A-NHEJ protein complex. Overexpression of MALAT1 was previously described as a poor prognostic marker for lung, breast, prostate, pancreatic cancers and glioma, as well as leukemia (Sun and Ma, 2019). Bone marrow plasma cells from patients with MGUS and MM were reported to express elevated levels of MALAT1 RNA (Hu et al., 2018). Degradation of the MALAT1 RNA by RNase H using anti-sense gapmer DNA oligos in MM cells stimulated poly-ADP-ribosylation of nuclear proteins. Anti-MALAT1 therapy combined with a PARP1 inhibitor or a proteasome inhibitor in MM cells displayed a synergistic effect *in vitro* (Hu et al., 2018).

### Epigenetic Therapy: Histone Modifiers and Hypomethylating Agents

Copy number alterations (CNA) are one of the most prominent genomic abnormalities in MM (Aktas Samur et al., 2019). The International Myeloma Working Group (IMWG) has designated 17p deletion and 1q21 gain (Chng et al., 2014) as poor prognostic CNA features in MM. Amplification of 1q21 is among the most frequent chromosomal aberrations in MM and is considered to be a high-risk genetic feature that is highly correlated with disease progression and drug resistance. These regions are known to contain a number of oncogenes including MCL1, IL6R, BCL9, CKS1B, ANP32E, ILF2, and ADAR1 which display synchronous amplification and deregulated expression (Marchesini et al., 2018). The primary mechanism of amplification of 1q21 in MM is the CN aberration known as “jumping translocation” 1q12 by which a duplication of the 1q12 peri-centromeric region translocates as a donor chromosome segment to one or more receptor chromosomes (Sawyer et al., 1998). Another possible cause of 1q amplification is KDM4A. KDM4A is a histone demethylase which binds to the BCL9 locus and induces replication and site-specific copy number gains of 1q12 and 1q21 (Sawyer et al., 2019). The *in vitro* modification of the 1q12 region by the DNA methylation inhibitor, 5-azacytidine, also provides evidence that site-specific hypomethylation of the 1q12 region can induce copy number gains of 1q21 and adjacent regions (Sawyer et al., 2019). This is an area of active investigation and epigenetic inhibitors may be developed as a treatment for MM.

Multiple myeloma SET domain/Wolf-Hirschhorn syndrome candidate 1 (MMSET/WHSC1) is a histone methyltransferase (HMT) which is overexpressed in t(4;14) MM. It has been shown that methylation of histones is linked to the ability of cells to undergo DNA damage repair (Michalak et al., 2019). Additionally, patients with t(4;14) MM often relapse following treatment with regimens that include DNA damage-inducing agents suggesting that MMSET may play a role in DNA damage repair and response. MMSET is required for efficient NHEJ as well as HR. Loss of MMSET led to loss of expression of several DNA repair proteins, as well as impaired recruitment of DNA repair proteins to sites of DNA DSBs. Following the addition of a DNA-damaging agent to MMSET-high cells, they repaired damaged DNA more efficiently and continued to propagate, whereas MMSET-low cells accumulated DNA damage and entered cell cycle arrest (Walker et al., 2014).

The epigenetic changes observed in MM suggest that clonal plasma cells may be susceptible to HDAC and DNA methyltransferases (DNMTs) inhibitors (Figure 1). By modulation of histones and non-histone proteins, HDACi are able to induce growth arrest and apoptosis, inhibit angiogenesis, and induce osteoblast maturation in MM cells. HDACi (Panobinostat, Vorinostat) were used as single agents or in combination with other anti-MM agents in several phase II and III clinical trials and showed promising clinical activity, although there was significant toxicity and agents allowing for a more selective targeting of HDAC are required. DNMTs inhibitors such as the hypomethylating agent, 5-azacitidine (5Aza-C), have also demonstrated cytotoxic activity against MM cells and exhibited synergistic effects in combination with bortezomib (Table 1). However, clinical studies have yet to confirm the therapeutic efficacy of DNMTs inhibitors in this disease. Of interest, microRNAs (miRNAs) have been implicated in the regulation of DNA methylation and may therefore represent a novel means to reprogramm the cellular methylome. Amodio et al. reported that mir-29b mimics target DNMT3A/3B and reduce global DNA methylation resulting in significant *in vivo* anti-tumor effects both alone, and in combination with demethylating agents.

Koduru et al. recently reported that activation-induced cytidine deaminase (AID) which is centrally involved in somatic hypermutation and class switch recombination may also be involved in mediating genomic instability in MM. AID-dependent genomic damage in MM cells involves receptor activator of nuclear factor kappa-B ligand (RANKL) signaling. Thus, targeted RANKL inhibition may interfere with this interaction and impair the development of further genomic instability. Of note, the AID gene expression signature corresponds to a slow rate of progression to MM in MGUS and SMM whereas the APOBEC profile corresponds to faster progression (Walker et al., 2015). Walker et al. reported in their 2015 Nature study that the APOBEC signature found in t(14;16) and t(14;20) MM patients is associated with a high mutation burden.

### Spindle Assembly Checkpoint and Microtubule Inhibitors

The Aurora kinase family consists of three serine/threonine protein kinases: Aurora kinase A, Aurora kinase B and Aurora kinase C (AURKA, AURKB, and AURKC) which are known to be key regulators of centrosome maturation, spindle assembly, chromosome segregation, and mitotic exit. Although these kinases share significant homology within their kinase domains and have 100% conserved ATP-binding sites (Kollareddy et al., 2008), AURKA and AURKB play essential roles in mitosis whereas AURKC is important for meiosis. AURKA promotes mitotic entry with phosphorylation of Polo-kinase 1 (Plk1) and consequent activation of the G2/M gatekeeper, cyclin B-CDK1 complex while AURKB has a crucial role in regulating the spindle checkpoint and cytokinesis (Goldenson and Crispino, 2015). In addition to its mitotic roles, AURKA acts as a transcription factor in cancer cells, promotes NHEJ repair by altering the expression and activity of genes involved in HR and plays a role in the maintenance of mitochondrial function (Do et al., 2017; Bertolin et al., 2018). Because the functions of AURK are fundamental to cell viability and their over-expression is associated with centrosome amplification and genomic instability, they represent potential targets for cancer therapy including in MM. There are several AURK inhibitors which have been evaluated pre-clinically or in clinical trials for the treatment of MM. Pan-AURK inhibitors including VX-680, danusertib (PHA-680632), ENMD-2076 and AT9283 which act against all aurora kinases have displayed anti-myeloma effects, both *in vitro* and *in vivo* (Shi et al., 2007; Evans R. et al., 2008; Hose et al., 2009; Negri et al., 2009; Wang et al., 2010; Fletcher et al., 2011). Danusertib was assessed in a phase II trial in relapsed, refractory MM (RRMM) patients though the trial was stopped due to poor recruitment (Lind et al., 2019). ENMD-2076 was evaluated in a phase I trial of which the results are still pending and a phase II trial of AT9283 in RRMM fail to display clinical responses (Hay et al., 2016).

Alisertib (MLN8237) is a specific AURKA inhibitor which is able to induce G2/M cell cycle arrest, mitotic spindle abnormalities, senescence, and apoptosis in MM cells (Figure 1). Its anti-MM effect was confirmed *in vivo* (Görgün et al., 2010). Following pre-clinical results, an open-label phase I study investigated the dose-limiting toxicities, pharmacokinetics and anti-tumor activity of alisertib in patients with advanced hematological malignancies including MM (Table 1). This study demonstrated the preliminary anti-MM activity of Alisertib in RRMM patients and non-Hodgkin Lymphoma (Kelly et al., 2014). Another phase I open-label multicentre clinical trial was conducted to test the efficacy of alisertib in combination with bortezomib. At a median follow-up of 20.6 months, the overall response rate was 26.9%, thereby demonstrating the feasibility of this combination. However, a Phase II study is now required (Rosenthal et al., 2016). Barasertib (AZD1152) is a selective AURKB inhibitor that induced apoptosis in MM cell lines and in CD138-selected plasma cells from myeloma patients. Although it was toxic to CD138-selected bone marrow cells from the same patients, barasertib was reported to suppress tumor growth and induce cell death with an acceptable safety profile in a murine myeloma xenograft model (Evans R. P. et al., 2008).

Kinesin spindle proteins (KSPs), members of the large kinesin superfamily of cytoskeletal motor proteins, play an essential role in cell division by promoting the segregation of centrosomes and by maintaining bipolar spindle assembly via ATP hydrolysis (Sarli and Giannis, 2008). Furthermore, KSPs have anti-apoptotic properties via cell survival protein myeloid leukemia sequence 1 (Mcl-1) which is generally over-expressed in MM cells and the levels of which correlate with a poor prognosis (Wuillème-Toumi et al., 2005; Tunquist et al., 2010). Inhibition of KSP activity leads to metaphase arrest as a result of the formation of aberrant monopolar as opposed to bipolar spindles and by impairing the segregation of centrosomes (Stern and Murray, 2001) (Figure 1). To date, filanesib (ARRY-520), a selective KSP inhibitor, has been evaluated in six clinical trials performed in RRMM patients, either as a single agent or in combination with other drugs which are used for the treatment of MM (Table 1). Two phase II studies of filanesib, one with and one without the granulocyte-colony stimulating factor (G-CSF), filgrastim, have been performed. In one of these trials, low dose dexamethasone was added in patients who had had prior alkylator therapy and who were refractory to lenalidomide, bortezomib and dexamethasone. This study showed response rates of 16% (filanesib) and 15% (filanesib + dexamethasone, evidence of some efficacy in heavily pre-treated, triple−refractory patients (Shah et al., 2017). In this study, low levels of alpha-1 acid glycoprotein, an acute-phase protein which can bind filanesib, was reported to be a useful biomarker that correlated with clinical response (Shah et al., 2017). Based on encouraging results in preclinical *in vivo* studies in which filanesib was combined with pomalidomide, the anti-myeloma efficacy of the triplet combination of filanesib, pomalidomide and dexamethasone was evaluated both *in vitro* and *in vivo* (Hernández-García et al., 2017). This combination revealed strong synergy and resulted in an increase in the number of monopolar spindles and level of BAX, cell cycle arrest in mitosis and subsequent apoptosis (Hernández-García et al., 2017). In light of these results, the Spanish Myeloma Group conducted a clinical trial (POMDEFIL) of this triplet combination in RRMM patients to assess safety and efficacy. Another triplet combination of filanesib, bortezomib, and dexamethasone was assessed in a phase I trial conducted in patients with RRMM and showed some durable responses in RRMM patients (Chari et al., 2016). In a phase I study, the combination of filanesib, carfilzomib, and dexamethasone was demonstrated to be safe and to have few side effects though the efficacy of this combination was limited (Lee et al., 2019). The dose-limiting toxicity of filanesib in these cited studies was neutropenia. In general, filanesib merits further investigation in this patient population (Lorusso et al., 2015).

### WNT/B-Catenin Inhibitors

The Wnt signaling pathway is constitutively activated in MM, thereby stimulating cell proliferation (Schmeel et al., 2013). This signaling pathway is therefore a potential target (Table 1). Thalidomide is the first drug that was found to inhibit this pathway (Figure 1). Recently, a study identified CGK012 as a small-molecule inhibitor of Wnt/β-catenin signaling which promotes β-catenin phosphorylation/degradation and repression of the expression of β-catenin-dependent genes, thereby inhibiting the proliferation of MM cells (Choi et al., 2017). Another study described another small molecule inhibitor, BC2059, which showed synergistic activity with bortezomib (Savvidou et al., 2017). In addition, an antifungal drug, griseofulvin, has been shown to induce apoptosis of myeloma and lymphoma cells *in vitro* and *in vivo* (Kim et al., 2011). Its exact mechanism of action is unknown but may involve centrosome de-clustering (Ferguson et al., 2015).

### NER Inhibition

The NER pathway recognizes DNA damage induced by ultraviolet light, tobacco, alkylating agents or DNA crosslinks and repairs them (Alekseev and Coin, 2015). NER activity varies in MM. One study found that cell lines with high NER activity tend to be resistant to melphalan. In addition, excision repair cross-complementation group 3 (ERCC3) overexpression increased resistance to melphalan confirming the function of NER in conferring resistance to alkylating agents. ERCC3 knock-down/NER deficiency led to a significant increase in sensitivity to melphalan (Szalat et al., 2018). Interestingly, spironolactone has been found to be a potent inhibitor of NER (Alekseev et al., 2014) Szalat et al. (2018) found that NER inhibition with spironolactone was able to restore melphalan sensitivity in MM cell lines.

### Telomerase Inhibitors

Telomere length is highly prognostic in MM (Hyatt et al., 2017). In healthy cells, dysfunctional telomeres and abnormal chromosomal structures induce a p53-mediated DNA damage response and activate the p16/pRB tumor suppressor pathway. Tumor supressor genes halt cell cycle progression, initiate senescence and prevent the propagation of abnormal chromosomes. The telomeres of cells that bypass senescence continue to shorten, leading to the evolution of complex karyotypes (MacKenzie et al., 2015). In one study, critically short telomeres were found to be fusogenic, triggering the formation of unstable structures such as dicentric or ring chromosomes. Thus, continued telomere dysfunction induces the preservation of abnormal chromosomes by breakage-fusion-bridge (BFB) cycles which are initiated by fused chromatids (Counter et al., 1992). The BFB cycle was reported to be associated with CIN development and peri-centromeric instability in MM (Sawyer et al., 2009). The most promising drug to specifically target telomerase is GRN163L, a synthetic lipid-conjugated 13-mer N3→P5 thio-phosphoramidate deoxyribo-oligonucleotide that blocks the template zone of telomerase and has potential antineoplastic activity (Schrank et al., 2018) (Figure 1). A study using myeloma cell lines found that prominent inhibition of telomerase activity with GRN1613L led to a reduction in viability to < 5% of baseline levels over a period of three to 5 weeks (Shammas et al., 2008) (Table 1).

## Conclusion

There are currently several small molecule inhibitors targeting chromosomal instability such as PARP, Akt, Aurora kinase and spindle kinase inhibitors which have been tested in mouse models and in early phase I/II trials. ATM, ATR kinase inhibitors and DNA helicase inhibitors are also promising novel agents. These drugs have been evaluated in patients with highly refractory MM and although not effective as monotherapy, show strong synergy when combined with DNA-damaging agents such as radiotherapy, platinum derivatives, immunomodulators and proteasome inhibitors.

This emerging field of genomic instability in myeloma precursor states is discussed further in other chapters of this special topic issue and may in future influence our approach to asymptomatic myeloma.

## Author Contributions

MB designed the outline of the manuscript. All authors performed the literature research and wrote the manuscript.

## Conflict of Interest

The authors declare that the research was conducted in the absence of any commercial or financial relationships that could be construed as a potential conflict of interest.

## References

[B1] Affer M. Chesi M. Chen W. D. Keats J. J. Demchenko Y. N. Tamizhmani K. (2014). Promiscuous MYC locus rearrangements hijack enhancers but mostly super-enhancers to dysregulate MYC expression in multiple myeloma. *Leukemia* 28 1725–1735. 10.1038/leu.2014.70 24518206 PMC4126852

[B2] Aktas Samur A. Minvielle S. Shammas M. Fulciniti M. Magrangeas F. Richardson P. G. (2019). Deciphering the chronology of copy number alterations in multiple myeloma. *Blood Cancer J.* 9:39. 10.1038/s41408-019-0199-3 30914633 PMC6435669

[B3] Alagpulinsa D. A. Ayyadevara S. Yaccoby S. Shmookler Reis R. J. (2016). A cyclin-dependent kinase inhibitor, dinaciclib, impairs homologous recombination and sensitizes multiple myeloma cells to PARP inhibition. *Mol. Cancer Ther.* 15 241–250. 10.1158/1535-7163.MCT-15-0660 26719576 PMC4747838

[B4] Alekseev S. Ayadi M. Brino L. Egly J. M. Larsen A. K. Coin F. (2014). A small molecule screen identifies an inhibitor of DNA repair inducing the degradation of TFIIH and the Chemosensitization of tumor cells to platinum. *Chem. Biol.* 21 398–407. 10.1016/j.chembiol.2013.12.014 24508195

[B5] Alekseev S. Coin F. (2015). Orchestral maneuvers at the damaged sites in nucleotide excision repair. *Cell. Mol. Life Sci.* 72 2177–2186. 10.1007/s00018-015-1859-5 25681868 PMC11113351

[B6] Amodio N. Leotta M. Bellizzi D. Di Martino M. T. D’Aquila P. Lionetti M. (2012). DNA-demethylating and anti-tumor activity of synthetic miR-29b mimics in multiple myeloma. *Oncotarget* 10 1246–1258. 10.18632/oncotarget.675 23100393 PMC3717964

[B7] Bertolin G. Bulteau A. L. Alves-Guerra M. C. Burel A. Lavault M. T. Gavard O. (2018). Aurora kinase a localises to mitochondria to control organelle dynamics and energy production. *eLife* 7:e38111. 10.7554/eLife.38111 30070631 PMC6140714

[B8] Chari A. Htut M. Zonder J. A. Fay J. W. Jakubowiak A. J. Levy J. B. (2016). No title. *Cancer* 122 3327–3335. 27433944 10.1002/cncr.30174 PMC6857452

[B9] Chng W. J. Ahmann G. J. Henderson K. Santana-Davila R. Greipp P. R. Gertz M. A. (2006). Clinical implication of centrosome amplification in plasma cell neoplasm. *Blood* 107 3669–3675. 10.1182/blood-2005-09-3810 16373658 PMC1895774

[B10] Chng W. J. Braggio E. Mulligan G. Bryant B. Remstein E. Valdez R. (2008). The centrosome index is a powerful prognostic marker in myeloma and identifies a cohort of patients that might benefit from aurora kinase inhibition. *Blood* 111 1603–1609. 10.1182/blood-2007-06-097774 18006703

[B11] Chng W. J. Dispenzieri A. Chim C. S. Fonseca R. Goldschmidt H. Lentzsch S. (2014). IMWG consensus on risk stratification in multiple myeloma. *Leukemia* 28 269–277. 10.1038/leu.2013.247 23974982

[B12] Choi P. J. Yuseok O. Her J. H. Yun E. Song G. Y. Oh S. (2017). Anti-proliferative activity of CGK012 against multiple myeloma cells via Wnt/β-catenin signaling attenuation. *Leuk. Res.* 60 103–108. 10.1016/j.leukres.2017.07.001 28772205

[B13] Counter C. M. Avilion A. A. LeFeuvre C. E. Stewart N. G. Greider C. W. Harley C. B. (1992). Telomere shortening associated with chromosome instability is arrested in immortal cells which express telomerase activity. *EMBO J.* 11 1921–1929. 10.1002/j.1460-2075.1992.tb05245.x 1582420 PMC556651

[B14] Dementyeva E. Kryukov F. Kubiczkova L. Nemec P. Sevcikova S. Ihnatova I. (2013). Clinical implication of centrosome amplification and expression of centrosomal functional genes in multiple myeloma. *J. Transl. Med.* 11:77. 10.1186/1479-5876-11-77 23522059 PMC3615957

[B15] Dementyeva E. Nemec P. Kryukov F. Muthu Raja K. R. Smetana J. Zaoralova R. (2010). Centrosome amplification as a possible marker of mitotic disruptions and cellular carcinogenesis in multiple myeloma. *Leuk. Res.* 34 1007–1011. 10.1016/j.leukres.2009.12.018 20096458

[B16] Do T. V. Hirst J. Hyter S. Roby K. F. Godwin A. K. (2017). Aurora A kinase regulates non-homologous end-joining and poly(ADP-ribose) polymerase function in ovarian carcinoma cells. *Oncotarget* 8 50376–50392. 10.18632/oncotarget.18970 28881569 PMC5584138

[B17] Evans R. Naber C. Steffler T. Checkland T. Keats J. Maxwell C. (2008). Aurora A kinase RNAi and small molecule inhibition of Aurora kinases with VE-465 induce apoptotic death in multiple myeloma cells. *Leuk. Lymphoma* 49 559–569. 10.1080/10428190701824544 18297535

[B18] Evans R. P. Naber C. Steffler T. Checkland T. Maxwell C. A. Keats J. J. (2008). The selective Aurora B kinase inhibitor AZD1152 is a potential new treatment for multiple myeloma. *Br. J. Haematol.* 140 295–302. 10.1111/j.1365-2141.2007.06913.x 18076711

[B19] Ferguson L. R. Chen H. Collins A. R. Connell M. Damia G. Dasgupta S. (2015). Genomic instability in human cancer: molecular insights and opportunities for therapeutic attack and prevention through diet and nutrition. *Semin. Cancer Biol.* 35 S5–S24. 10.1016/j.semcancer.2015.03.005 25869442 PMC4600419

[B20] Fletcher G. C. Brokx R. D. Denny T. A. Hembrough T. A. Plum S. M. Fogler W. E. (2011). ENMD-2076 is an orally active kinase inhibitor with antiangiogenic and antiproliferative mechanisms of action. *Mol. Cancer Ther.* 10 126–137. 10.1158/1535-7163.MCT-10-0574 21177375

[B21] Goldenson B. Crispino J. D. (2015). The aurora kinases in cell cycle and leukemia. *Oncogene* 34 537–545. 10.1038/onc.2014.14 24632603 PMC4167158

[B22] Görgün G. Calabrese E. Hideshima T. Ecsedy J. Perrone G. Mani M. (2010). Anovel Aurora-A kinase inhibitor MLN8237 induces cytotoxicity and cell-cycle arrest in multiple myeloma. *Blood* 115 5202–5213. 10.1182/blood-2009-12-259523 20382844 PMC2892955

[B23] Gourzones-Dmitriev C. Kassambara A. Sahota S. Rème T. Moreaux J. Bourquard P. (2013). DNA repair pathways in human multiple myeloma: role in oncogenesis and potential targets for treatment. *Cell Cycle* 12 2760–2773. 10.4161/cc.25951 23966156 PMC3899190

[B24] Hay A. E. Murugesan A. Dipasquale A. M. Kouroukis T. Sandhu I. Kukreti V. (2016). A phase II study of AT9283, an aurora kinase inhibitor, in patients with relapsed or refractory multiple myeloma: NCIC clinical trials group IND.191. *Leuk. Lymphoma* 57 1463–1466. 10.3109/10428194.2015.1091927 26376958

[B25] Hernández-García S. San-Segundo L. González-Méndez L. Corchete L. A. Misiewicz-Krzeminska I. Martín-Sánchez M. (2017). The kinesin spindle protein inhibitor filanesib enhances the activity of pomalidomide and dexamethasone in multiple myeloma. *Haematologica* 102 2113–2124. 10.3324/haematol.2017.168666 28860344 PMC5709111

[B26] Hose D. Rème T. Meissner T. Moreaux J. Seckinger A. Lewis J. (2009). Inhibition of aurora kinases for tailored risk-adapted treatment of multiple myeloma. *Blood* 113 4331–4340. 10.1182/blood-2008-09-178350 19171872 PMC2700334

[B27] Hu Y. Lin J. Fang H. Fang J. Li C. Chen W. (2018). Targeting the MALAT1/PARP1/LIG3 complex induces DNA damage and apoptosis in multiple myeloma. *Leukemia* 32 2250–2262. 10.1038/s41375-018-0104-2 29632340 PMC6151178

[B28] Hyatt S. Jones R. E. Heppel N. H. Grimstead J. W. Fegan C. Jackson G. H. (2017). Telomere length is a critical determinant for survival in multiple myeloma. *Br. J. Haematol.* 178 94–98. 10.1111/bjh.14643 28342200

[B29] Kassambara A. Gourzones-Dmitriev C. Sahota S. Rème T. Moreaux J. Goldschmidt H. (2015). A DNA repair pathway score predicts survival in human multiple myeloma: the potential for therapeutic strategy. *Oncotarget* 5 2487–2498. 24809299 10.18632/oncotarget.1740 PMC4058021

[B30] Kelly K. R. Shea T. C. Goy A. Berdeja J. G. Reeder C. B. McDonagh K. T. (2014). Phase I study of MLN8237 - Investigational Aurora A kinase inhibitor - In relapsed/refractory multiple myeloma, non-Hodgkin lymphoma and chronic lymphocytic leukemia. *Invest. New Drugs* 32 489–499. 10.1007/s10637-013-0050-9 24352795 PMC4045308

[B31] Kim Y. Alpmann P. Blaum-Feder S. Krämer S. Endo T. Lu D. (2011). In vivo efficacy of griseofulvin against multiple myeloma. *Leuk. Res.* 35 1070–1073. 10.1016/j.leukres.2010.10.008 21112630

[B32] Kollareddy M. Dzubak P. Zheleva D. Hajduch M. (2008). Aurora kinases: structure, functions and their association with cancer. *Biomed. Pap. Med. Fac. Univ. Palacky Olomouc Czech. Repub.* 152 27–33. 10.5507/bp.2008.004 18795071

[B33] Lee H. C. Shah J. J. Feng L. Manasanch E. E. Lu R. Morphey A. (2019). A phase 1 study of filanesib, carfilzomib, and dexamethasone in patients with relapsed and/or refractory multiple myeloma. *Blood Cancer J.* 9:80. 10.1038/s41408-019-0240-6 PMC6773683 31575851

[B34] Lind J. Czernilofsky F. Vallet S. P. K. (2019). No title. *Expert Opin. Emerg. Drugs* 24 133–152. 31327278 10.1080/14728214.2019.1647165

[B35] Lord C. J. McDonald S. Swift S. Turner N. C. Ashworth A. (2008). A high-throughput RNA interference screen for DNA repair determinants of PARP inhibitor sensitivity. *DNA Repair.* 7 2010–2019. 10.1016/j.dnarep.2008.08.014 18832051

[B36] Lorusso P. M. Goncalves P. H. Casetta L. Carter J. A. Litwiler K. Roseberry D. (2015). First-in-human phase 1 study of filanesib (ARRY-520), a kinesin spindle protein inhibitor, in patients with advanced solid tumors. *Invest. New Drugs* 33 440–449. 10.1007/s10637-015-0211-0 25684345

[B37] MacKenzie K. L. Halicka D. Amedei A. Aquilano K. Ashraf S. S. Firestone G. L. (2015). Therapeutic targeting of replicative immortality. *Semin. Cancer Biol.* 35 S104–S128. 10.1016/j.semcancer.2015.03.007 25869441 PMC4600408

[B38] Maes A. Menu E. de Veirman K. Maes K. Vanderkerken K. de Bruyne E. (2017). The therapeutic potential of cell cycle targeting in multiple myeloma. *Oncotarget* 8 90501–90520. 10.18632/oncotarget.18765 29163849 PMC5685770

[B39] Marchesini M. Ogoti Y. Fiorini E. Samur A. A. Nezi L. D’Anca M. (2018). ILF2 is a regulator of RNA splicing and DNA damage response in 1q21-amplified multiple myeloma. *Cancer Cell* 32 88–100. 10.1016/j.ccell.2017.05.011 28669490 PMC5593798

[B40] Michalak E. M. Burr M. L. Bannister A. J. Dawson M. A. (2019). The roles of DNA, RNA and histone methylation in ageing and cancer. *Nat. Rev. Mol. Cell Biol.* 20 573–589. 10.1038/s41580-019-0143-1 31270442

[B41] Negri J. M. McMillin D. W. Delmore J. Mitsiades N. Hayden P. Klippel S. (2009). In vitro anti-myeloma activity of the Aurora kinase inhibitor VE-465. *Br. J. Haematol.* 147 672–676. 10.1111/j.1365-2141.2009.07891.x 19751238 PMC3672411

[B42] Neri P. Bahlis N. J. (2013). Genomic instability in multiple myeloma: mechanisms and therapeutic implications. *Expert Opin. Biol. Ther.* 13 S69–S82. 10.1517/14712598.2013.814637 23782016

[B43] Neri P. Ren L. Gratton K. Stebner E. Johnson J. Klimowicz A. (2011). Bortezomib-induced ‘BRCAness’ sensitizes multiple myeloma cells to PARP inhibitors. *Blood* 118 6368–6379. 10.1182/blood-2011-06-363911 21917757 PMC4348156

[B44] Patel P. R. Senyuk V. Rodriguez N. S. Oh A. L. Bonetti E. Mahmud D. (2019). Synergistic cytotoxic effect of busulfan and the PARP inhibitor veliparib in myeloproliferative neoplasms. *Biol. Blood Marrow Transplant.* 25 855–860. 10.1016/j.bbmt.2018.12.841 30615982

[B45] Quintyne N. J. Reing J. E. Hoffelder D. R. Gollin S. M. Saunders W. S. (2005). Spindle multipolarity is prevented by centrosomal clustering. *Science* 307 127–129. 10.1126/science.1104905 15637283

[B46] Rosenthal A. Kumar S. Hofmeister C. Laubach J. Vij R. Dueck A. (2016). A phase Ib study of the combination of the aurora kinase inhibitor alisertib (MLN8237) and bortezomib in relapsed multiple myeloma. *Br. J. Haematol.* 174 323–325. 10.1111/bjh.13765 26403323 PMC4820359

[B47] Sarli V. Giannis A. (2008). Targeting the kinesin spindle protein: basic principles and clinical implications. *Clin. Cancer Res.* 14 7583–7587. 10.1158/1078-0432.CCR-08-0120 19047082

[B48] Savvidou I. Khong T. Cuddihy A. McLean C. Horrigan S. Spencer A. (2017). β-Catenin inhibitor BC2059 is efficacious as monotherapy or in combination with proteasome inhibitor bortezomib in multiple myeloma. *Mol. Cancer Ther.* 16 1765–1778. 10.1158/1535-7163.MCT-16-0624 28500235

[B49] Sawyer J. R. Tian E. Heuck J. Johann D. J. Epstein J. Swanson C. M. (2015). Evidence of an epigenetic origin for high-risk 1q21 copy number aberrations in multiple myeloma. *Blood* 125 3756–3759. 10.1182/blood-2015-03-632075 25943786 PMC4463736

[B50] Sawyer J. R. Tian E. Thomas E. Koller M. Stangeby C. Sammartino G. (2009). Evidence for a novel mechanism for gene amplification in multiple myeloma: 1q12 pericentromeric heterochromatin mediates breakage-fusion-bridge cycles of a 1q12~23 amplicon. *Br. J. Haematol.* 147 484–494. 10.1111/j.1365-2141.2009.07869.x 19744130 PMC3738438

[B51] Sawyer J. R. Tian E. Walker B. A. Wardell C. Lukacs J. L. Sammartino G. (2019). An acquired high-risk chromosome instability phenotype in multiple myeloma: jumping 1q syndrome. *Blood Cancer J.* 9:62. 10.1038/s41408-019-0226-4 31399558 PMC6689064

[B52] Sawyer J. R. Tricot G. Mattox S. Jagannath S. Barlogie B. (1998). Jumping translocations of chromosome 1q in multiple myeloma: evidence for a mechanism involving decondensation of pericentromeric heterochromatin. *Blood* 91 1732–1741. 10.1182/blood.v91.5.1732.1732_1732_1741 9473240

[B53] Schmeel L. C. Schmeel F. C. Kim Y. Endo T. Lu D. Schmidt-Wolf I. G. H. (2013). Targeting the Wnt/beta-catenin pathway in multiple myeloma. *Anticancer Res.* 33 4719–4726. 24222106

[B54] Schrank Z. Khan N. Osude C. Singh S. Miller R. J. Merrick C. (2018). Oligonucleotides targeting telomeres and telomerase in cancer. *Molecules* 23:2267. 10.3390/molecules23092267 30189661 PMC6225148

[B55] Shah J. J. Kaufman J. L. Zonder J. A. Cohen A. D. Bensinger W. I. Hilder B. W. (2017). No title. *Cancer* 123 4617–4630. 28817190 10.1002/cncr.30892 PMC5856158

[B56] Shaheen M. Allen C. Nickoloff J. A. Hromas R. (2011). Synthetic lethality: exploiting the addiction of cancer to DNA repair. *Blood* 117 6074–6082. 10.1182/blood-2011-01-313734 21441464

[B57] Shammas M. A. Koley H. Bertheau R. C. Neri P. Fulciniti M. Tassone P. (2008). Telomerase inhibitor GRN163L inhibits myeloma cell growth in vitro and in vivo. *Leukemia* 22 1410–1418. 10.1038/leu.2008.81 18449204 PMC3155939

[B58] Shi Y. Reiman T. Li W. Maxwell C. A. Sen S. Pilarski L. (2007). Targeting aurora kinases as therapy in multiple myeloma. *Blood* 109 3915–3921. 10.1182/blood-2006-07-037671 17213289 PMC1874561

[B59] Stern B. M. Murray A. W. (2001). Lack of tension at kinetochores activates the spindle checkpoint in budding yeast. *Curr. Biol.* 11 1462–1467. 10.1016/s0960-9822(01)00451-1 11566107

[B60] Sun Y. Ma L. (2019). New insights into long non-coding RNA *MALAT1* in cancer and metastasis. *Cancers* 11:216. 10.3390/cancers11020216 30781877 PMC6406606

[B61] Szalat R. Samur M. K. Fulciniti M. Lopez M. Nanjappa P. Cleynen A. (2018). Nucleotide excision repair is a potential therapeutic target in multiple myeloma. *Leukemia* 32 111–119. 10.1038/leu.2017.182 28588253 PMC5720937

[B62] Tunquist B. J. Woessner R. D. Walker D. H. (2010). Mcl-1 stability determines mitotic cell fate of human multiple myeloma tumor cells treated with the kinesin spindle protein inhibitor ARRY-520. *Mol. Cancer Ther.* 9 2046–2056. 10.1158/1535-7163.MCT-10-0033 20571074

[B63] Walker B. A. Wardell C. P. Brioli A. Boyle E. Kaiser M. F. Begum D. B. (2014). Translocations at 8q24 juxtapose MYC with genes that harbor superenhancers resulting in overexpression and poor prognosis in myeloma patients. *Blood Cancer J.* 4 e191–e197. 10.1038/bcj.2014.13 24632883 PMC3972699

[B64] Walker B. A. Wardell C. P. Murison A. Boyle E. M. Begum D. B. Dahir N. M. (2015). APOBEC family mutational signatures are associated with poor prognosis translocations in multiple myeloma. *Nat. Commun.* 6:6997. 10.1038/ncomms7997 25904160 PMC4568299

[B65] Wang X. Sinn A. L. Pollok K. Sandusky G. Zhang S. Chen L. (2010). Preclinical activity of a novel multiple tyrosine kinase and aurora kinase inhibitor, ENMD-2076, against multiple myeloma. *Br. J. Haematol.* 150 313–325. 10.1111/j.1365-2141.2010.08248.x 20560971

[B66] Wuillème-Toumi S. Robillard N. Gomez P. Moreau P. Le Gouill S. Avet-Loiseau H. (2005). Mcl-1 is overexpressed in multiple myeloma and associated with relapse and shorter survival. *Leukemia* 19 1248–1252. 10.1038/sj.leu.2403784 15902294

